# A broader outlook is required to stage and classify pituitary neoplasms for patient care

**DOI:** 10.1111/bpa.13321

**Published:** 2024-11-23

**Authors:** Shlomo Melmed, Maria Fleseriu, John A. H. Wass, Ken K. Y. Ho

**Affiliations:** ^1^ Department of Medicine Cedars‐Sinai Medical Center Los Angeles California USA; ^2^ Departments of Medicine and Neurological Surgery Oregon Health and Science University Portland Oregon USA; ^3^ Department of Endocrinology Churchill Hospital Oxford UK; ^4^ The Garvan Institute of Medical Research Sydney New South Wales Australia

To the Editor:

Synthesis of the current literature in a robust review is important for advancing science as it provides updated objective evidence‐based information to the reader [1]. Strong and fair reviews should serve as a credible resource for the reader, marshaling strengths and weaknesses of available evidence on a particular topic. Evidence sources should include all relevant current literature, particularly publications in high‐impact journals. The review should thus offer a balanced appraisal of areas of agreement and controversy, highlight unanswered questions, and present opportunities for future research.

Regrettably, the Invited Review from Villa et al. [2] on the grading and staging system for pituitary neuroendocrine tumors (PitNETs) based on the WHO Classification of Endocrine and Neuroendocrine Tumors falls short of these criteria for a fair and balanced review. The authors contend that the review “illustrates the main issues involved in establishing a grading and a staging system, as well as alternative systems.” However, it does not mention a body of recent literature devoted to the WHO's controversial classification of pituitary adenomas as PitNETs [3, 4]. They advocate using a histo‐molecular approach but do not address a consensus recommendation from the international multidisciplinary PANOMEN Workshop for a comprehensive pituitary adenoma classification embodying histo‐molecular markers [5].

Unambiguous communication of medical advances to the scientist and practitioner should aim to enhance clinical care. However, the utility of new classification proposals may be impaired by subjective opinions when not subjected to informed peer review, and may harm the foundation of medical communication. Impartial criteria should be applied as objective determinants of a new classification. These may include interdisciplinary clinical relevance, as well as universal physician and patient acceptance.

The PANOMEN classification was proposed by members of professional societies representing clinical disciplines who care for patients with these lesions, including the Endocrine Society, Pituitary Society, European Society of Endocrinology, International Society of Pituitary Surgeons, American Association of Clinical Endocrinology, and US and Canadian Academy of Pathology, and endorsed by patient support organizations [5]. Scientific peer‐reviewed evidence was collected and underwent robust multidisciplinary analysis and discussion prior to peer review and publication. These elements are glaringly lacking in the formulation of the WHO classification. Indeed, a recent Commentary [6] highlights the need for this type of multidisciplinary approach and points to flaws in the WHO classification that could engender patient mismanagement through misuse of inappropriate nomenclature in the clinic.

Unlike the histology‐based WHO classification, the PANOMEN classification includes weighted scoring for disease phenotype at clinical presentation, adenoma secretory status, presence of pituitary failure, adenoma size and degree of invasion as determined by pituitary‐directed MRI, presence of post‐operative residual tissue determined by MRI, comprehensive immuno‐histopathology of resected tissue (if available), and the presence of a familial syndrome (e.g., MEN1). The proposed model does not include patient age or sex or somatic mutation information, as these factors have not yet been determined to impact disease outcome [5].

This distinction in the basis of the classification system between WHO and PANOMEN is critical. The vast majority of pituitary adenomas are indolent, with only 1 in 1000 causing symptoms (Figure 1) [7]. Among these, more than 50% are not surgically resected and no histologic diagnosis is ever made. Only about 0.2% of surgically resected pituitary adenomas are malignant, representing a prevalence of malignancy of 1 per million of pituitary adenomas.

**FIGURE 1:**
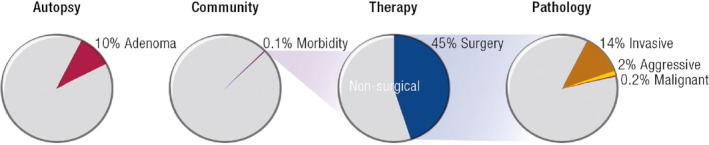
Epidemiology of pituitary adenomas. Reprinted with permission from Melmed et al. [7].

Villa et al. seem surprised by the “unexpectedly” low rate of histopathologically confirmed pituitary adenomas in the 2023 Central Brain Tumor Registry of the United States. They further note the very low incidence of malignant tumors in this Registry reflects underestimation and inconsistent application of classification and grading. They suggest both of these concerns could be ameliorated once the PitNET nomenclature is widely used. In fact, the numbers simply reflect that the majority of pituitary adenomas are benign, are not surgically resected, and will never receive a histopathologic diagnosis. It is therefore unclear what a change of nomenclature would accomplish in practice. Indeed, it has been noted that it offers no advantage to pituitary neurosurgical workflows [8].

The authors mention only parenthetically that classification of these adenomas as neuroendocrine tumors assigns them oncologic grades within the International Classification of Diseases for Oncology. Yet, this oncologic grading engenders a major adverse impact on patient anxiety and constrains their ability to obtain health, disability, and life insurance, and may impede employment opportunities. Such a label may also generate unnecessary oncology evaluation and testing.

The authors correctly note that a “classification is intended to aid correct diagnosis” and is not intended to “estimate prognosis or predict response to therapy.” Yet, the WHO classification erroneously equates a pathologic description with a clinical phenotype that the authors describe in their workflow schema as having “malignant potential.” Accordingly, the schema omits the overwhelming majority of pituitary adenomas that are benign and not commonly resected—prolactinomas, small nonfunctioning microadenomas, and some slowly growing nonfunctioning macroadenomas—and these commonly encountered lesions would therefore be excluded from the proposed clinical‐histo‐molecular workflow. Curiously, authoritative workflows and guideline recommendations recently published for prolactinoma, Cushing disease, and acromegaly [9, 10, 11] are not mentioned.

We respectfully urge *Brain Pathology* editors to offer their readers an objective review of the evidence surrounding the challenge of developing a comprehensive pituitary adenoma classification that consolidates clinical, biochemical, imaging, and omics as disease outcome biomarkers. Common sense dictates that our valued pathology colleagues work in tandem with our medical and surgical clinicians [6]. Our patients deserve nothing less.

## CONFLICT OF INTEREST STATEMENT

The authors declare no conflicts of interest.

## Data Availability

Data sharing is not applicable to this article as no new data were created or analyzed in this study.
